# Comparison of the clinical courses and chemotherapy outcomes in metastatic colorectal cancer patients with and without active *Mycobacterium tuberculosis* or *Mycobacterium kansasii* infection: a retrospective study

**DOI:** 10.1186/1471-2407-14-770

**Published:** 2014-10-18

**Authors:** Tomonori Hirashima, Takayuki Nagai, Hironori Shigeoka, Yoshitaka Tamura, Hiroko Yoshida, Kunimitsu Kawahara, Yoko Kondoh, Kenichi Sakai, Shoji Hashimoto, Makoto Fujishima, Takayuki Shiroyama, Motohiro Tamiya, Naoko Morishita, Hidekazu Suzuki, Norio Okamoto, Ichiro Kawase

**Affiliations:** Department of Thoracic Malignancy, Osaka Prefectural Medical Center for Respiratory and Allergic Diseases, 3-7-1 Habikino, Habikino City, Osaka, 583-8588 Japan; Department of Infectious Diseases, Osaka Prefectural Medical Center for Respiratory and Allergic Diseases, 3-7-1 Habikino, Habikino City, Osaka, 583-8588 Japan; Department of Gastroenterological and Breast Surgery, Osaka Prefectural Medical Center for Respiratory and Allergic Diseases, 3-7-1 Habikino, Habikino City, Osaka, 583-8588 Japan; Department of Clinical laboratory, Osaka Prefectural Medical Center for Respiratory and Allergic Diseases, 3-7-1 Habikino, Habikino City, Osaka, 583-8588 Japan; Department of Pharmacy, Osaka Prefectural Medical Center for Respiratory and Allergic Diseases, 3-7-1 Habikino, Habikino City, Osaka, 583-8588 Japan

**Keywords:** Metastatic colorectal cancer, *Mycobacterium tuberculosis*, *Mycobacterium kansasii*, Cancer chemotherapy, Mycobacterium infection

## Abstract

**Background:**

Although active *Mycobacterium tuberculosis* (MTB) or *Mycobacterium Kansasii* (MK) infection could be present in patients with metastatic colorectal cancer (m-CRC), no study is available on the clinical courses and chemotherapy outcomes of these patients. The present study therefore aimed to retrospectively examine whether m-CRC patients with and without active MTB or MK infection could receive cancer chemotherapy similarly.

**Methods:**

This study enrolled 30 m-CRC patients who received first-line chemotherapy between January 31, 2006 and January 31, 2013 at our institution, The clinical courses and tumor response of those with and without active MTB or MK infection were examined and compared.

**Results:**

Of 30 m-CRC patients, 6 had active MTB infection, 1 with active MK and the other 23 had neither MTB nor MK. No significant demographic differences were observed between patients with MTB or MK and those without. Chemotherapy response rates of all patients, those with MTB or MK, and those without were 40.0%, 28.6% and 43.5%, respectively. Among patients with MTB or MK, 1 treated with bevacizumab experienced grade-3 hemoptysis while others did not report any severe toxicity. Median survival time of all studied patients, those with MTB or MK, and those without was 26.3, 36.7 and 22.6 months, respectively. No significant difference in overall survival was observed between patients with MTB or MK and those without. Multivariate analysis revealed that performance status and liver metastasis were significant prognostic factors of overall survival (P = 0.004 and 0.030, respectively), whereas other factors, including MTB or MK infection, were not. In our study, all 7 patients with MTB or MK did not experience infection relapse during or after cancer chemotherapy.

**Conclusions:**

Our results indicate that m-CRC patients with MTB or MK should be able to safely and effectively continue cancer chemotherapy to subsequently achieve comparable survival duration to those without the infection if they receive proper MTB or MK treatment.

## Background

Colorectal cancer (CRC) is the third most commonly diagnosed cancer in males and the second in females in 2008 [1]. The disease incidence rates are rapidly increasing in parts of Eastern Asia including Korea and the urban area of China, Eastern Europe, and Brazil [2, 3]. Particularly, the rate in Japan has exceeded the peak value observed in countries where incidence rates are declining or stabilizing such as the United States, Canada, and Australia [2, 3].

*Mycobacterium tuberculosis* (MTB) is the second leading cause of death from an infectious disease worldwide [4]. In 2012, most MTB cases occurred in Asia (58%) and Africa (27%). The disease incidence and mortality rates in Japan are obviously higher than those in other developed countries in North America and Western Europe [4].

Our institution is established as a medical center for respiratory diseases such as lung cancer, chronic obstructive pulmonary diseases, bronchial asthma, interstitial lung diseases, and various pulmonary infections including MTB and non-tuberculosis mycobacterium (NTM). Therefore, patients with abnormal findings by chest radiography or computed tomography (CT), or suspicious MTB infection are often referred to us. As a result, our medical oncologists, in collaboration with other specialists, have administered cancer chemotherapy in metastatic colorectal cancer (m-CRC) patients with multiple lung metastases, respiratory diseases including bronchial asthma and chronic obstructive pulmonary diseases, and MTB or NTM infection.

Although active MTB or NTM infection could be present in patients with m-CRC, no study is available on the clinical courses and chemotherapy outcomes of these patients. In this present study, we first reported that if proper tuberculosis management was provided, m-CRC patients with active MTB could concurrently receive both cancer chemotherapy and MTB treatment safely and effectively.

## Methods

### Study approval

The present retrospective study was approved by the Institutional Review Board of the Osaka Prefectural Medical Center for Respiratory and Allergic Diseases on October 23, 2013 (approval number: 654).

### Patient selection

Patients with m-CRC who received first-line chemotherapy between January 31, 2006 and January 31, 2013 at our institution were included. Those receiving adjuvant chemotherapy or eligible for surgical treatments of lung metastasis or liver metastasis were excluded from this study.

### Clinical review

Clinical history of eligible patients was retrospectively reviewed. Baseline demographics information including sex, age, Eastern Cooperative Oncology Group performance status (PS), histology, and disease stage at the beginning of first-line chemotherapy was obtained for each patient. Furthermore, chemotherapy data including regimen, number of cycles and dose, and the start and stop date of each regimen were collected from clinical records or pharmacy database. At the time of first-line chemotherapy, the following data were available for all patients: a complete history and physical examination, surgical reports, colonoscopy findings, imaging investigations (chest radiography, abdomen radiography and CT, brain CT or magnetic resonance imaging, abdominal CT, and bone scintigraphy or positron emission tomography), pathologic reports, and blood test results.

### Serum carcinoembryonic antigen (CEA)

Serum CEA levels (ng/mL) were assessed for inoperable or relapsed CRC at the beginning of and after first-line chemotherapy per our institutional routine protocol. The upper limit of normal range was 5 ng/mL.

### Genetic testing for K-ras mutation

Tissue samples from enrolled patients were sent at the appropriate time to SRL, Inc. (Tokyo, Japan) for K-ras mutation analysis. The cost of such procedure is covered by the Japanese medical insurance system for m-CRC patients.

### Chemotherapeutic regimens

At our institution, intensive regimens for first-line chemotherapy included fluorouracil/leucovorin plus oxaliplatin (FOLFOX) [5], fluorouracil/leucovorin plus irinotecan (FOLFIRI) [6], or capecitabine plus oxaliplatin (XELOX) administered with or without bevacizumab (Bmab) [7–9] or anti-epidermal growth factor receptor antibody such as cetuximab (Cmab) [10, 11] and panitumumab (Pmab) [12]. Patients could also receive second-line chemotherapy with intensive regimens different from the first-line. Additionally, those with poor PS or sever complications received different regimens from the intensive ones for either first- or second-line chemotherapy. Treatment was discontinued in cases of disease progression, unacceptable side effects, or at a patient’s request.

### Best response to first-line chemotherapy

Response Evaluation Criteria in Solid Tumors (RECIST) [13] were used to evaluate treatment response during weekly discussion at our institution. Patients’ best response to first-line chemotherapy was collected from records of these meetings and physicians’ summaries. Based on RECIST criteria, a tumor response to cytotoxic agents was categorized as partial response (PR), stable disease (SD), or progressive disease (PD). A tumor response that was not assessable was noted as not evaluable (NE).

### MTB or Mycobacterium kansasii (MK) diagnosis

Sputum smears and cultures were routinely tested for acid-fast bacilli with 3-day consecutive sputa if the patient was producing sputum. For patients incapable of producing a sputum sample, common alternative sample sources for mycobacterium diagnosis included those from gastric washing or bronchoscopy. In most patients without a microbiological evaluation, mycobacterium diagnosis was assessed by chest CT imaging.

The preferred method for mycobacterium infection determination was fluorescence microscopy study and Ziehl-Neelsen staining of sputum smear samples [14], of which results were reported within 24 hours of testing. If a sputum smear was positive, polymerase chain reaction (PCR) or loop-mediated isothermal amplification of DNA [15] could be performed to distinguish MTB from other mycobacteria, and the result of such procedure was reported within 2 days. If sputum smear was negative or specimens other than sputum were obtained from a patient, a definitive diagnosis of mycobacterial infection was made by culturing mycobacterial organisms from such a specimen. Sputum specimens from smear-positive MTB patients were also cultured for drug sensitivity. Liquid media with Mycobacteria Growth Indicator Tube [16] and solid media with Ogawa-Kudoh method [17] were both used for mycobacterial culture. Quantitative drug susceptibility testing for MTB was performed by MTB-I® (Kyokuto Pharmaceutical Industrial Co., LTD, Tokyo) modified Minimum Inhibitory Concentration method [18].

### Treatment of MTB or MK infection

Treatment strategy for patients with MTB or NTM including MK infection was discussed weekly at our meetings on infectious diseases. m-CRC patients with MTB infection started cancer chemotherapy after being treated with appropriate MTB drugs according to the American Thoracic Society (ATS) and the Infectious Diseases Society of America (IDSA) guidelines [19] for approximately one and a half months. It was required that MTB bacillus was not multi-drug resistant in a sensitivity test for cancer chemotherapy to be initiated. Among patients with NTM, only those with MK were treated similar to MTB patients according to a modified regimen based on ATS and IDSA guidelines [20]. Thus, MTB patients received a standard treatment including two months of a drug combination containing isoniazid (H), rifampicin (R), ethambutol (E), and pyrazinamide (P) (2HREZ), followed by four months of daily H and R (4HR). Those with MK infection received 12HR to 18HR. However, as MTB patients with severe complication often expected a longer treatment than those without, those treated with cancer chemotherapy received long-term treatment for MTB.

### Follow-up MTB or MK culture

After MTB or MK treatment started, sputum culture was performed bi-weekly for the first three months. When results of two consecutive sputum cultures were negative, the procedure was conducted monthly until the completion of MTB or MK treatment.

### Definition of MTB or MK treatment success

MTB or MK treatment success was comprised of a cure and a treatment completion. A cure was defined as both the completion of a planned treatment and the negativity of two consecutive cultures, whereas a treatment completion was considered as the former alone.

### Statistical analysis

Survival time was defined as the time from the initiation date of fist-line chemotherapy to the date of death or the last follow-up. Survival data were updated on October 31, 2013. Survival rates were estimated by the Kaplan-Meier method [21]. Differences among survival curves were assessed using the log-rank test. Variables with a *P-*value of <0.05 on univariate analysis were included in the multivariate analysis with Cox’s regression model to identify independent predictors of survival. All analyses were conducted using the statistical software package R [22]. All comparisons with a *P* value of less than 0.05 were considered statistically significant.

## Results

### Patient characteristics

Thirty patients with inoperable or relapsed m-CRC who received fist-line chemotherapy at our institution between January 1, 2006 and 2013 January 31 were included in this study. Among these, 11 patients were complicated with respiratory diseases, including 6 with active MTB infection and 1 with active MK. Table 1 summarizes the characteristics of all patients (N = 30), those with MTB or MK infection (N = 7), and those without MTB or MK infection (N = 23). No differences in sex, age, PS, serum CEA levels, and K-ras status were observed between patients with MTB or MK infection and those without. Of 30 patients, 15 had recurrence after CRC surgery and 15 were diagnosed at stage IV of the disease. Of 15 stage IV patients, 7 underwent palliative surgery for stenosis or obstruction in the colon or rectum.

**Table 1:** **Patient demographics**

Variable	Overall	MTB or MK	
		(+)	(–)
Total	30	7	23
Sex (male/female)	20/10	5/2	15/8
Median age (range), years	69 (43–85)	60 (56–73)	74 (43–85)
Performance status (0–1/2/3–4)	19/5/6	5/0/2	14/5/4
Primary site (rectum/colon)	17/13	5/2	12/11
Stage (IV/relapse)	15/15	5/2	10/13
Number of metastatic organs (1/≥2)	13/17	5/2	8/15
Metastatic sites			
Lung	18	4	14
Liver	13	4	9
Peritoneum	8	1	7
Abdominal lymph node	5	1	4
Bone	5	0	5
Mediastinal lymph node	4	0	4
Others	2	0	2
Serum CEA (ng/mL) level			
≤5/>5	7/23	2/5	5/18
K-ras status (wild–type/mutated/unknown)	12/14/4	4/3/0	8/11/4
Respiratory diseases			
MTB or MK infection	6/1	6/1	–
COPD with oxygen inhalation	2	0	2
Others	2	0	2

CEA: carcinoembryonic antigen, MTB: *Mycobacterium tuberculosis*, MK: *Mycobacterium kansasii*, COPD: chronic obstructive pulmonary disease.

### Chemotherapy regimens and response to first-line chemotherapy

Table 2 presents the first-line chemotherapy, which was categorized into intensive regimens and non-intensive regimens, for all m-CRC patients (N = 30), those with active MTB or MK infection (N = 7), and those without (N = 23). Of 30 enrolled patients, 1 achieved CR; 11 had PR; 10 had SD, and 7 had PD. The response rates (RR) of all patients, those with active MTB or MK infection, and those without were 40.0%, 28.6% and 43.5%, respectively. Of 2 MTB or MK responders, 1 was treated with FOLFOX plus Cmab, while the other received FOLFOX. Among 10 responders without MTB/MK infection, 5 were treated with FOLFOX + Bmab, 2 with FOLFOX, and 1 each with FOLFIRI alone, FOLFIRI + Bmab, or FOLFIRI + Pmab. None of the 4 patients who received non-intensive regimens achieved PR or CR.

**Table 2:** **Chemotherapy regimens, number of cycles and patients’ response to first-line chemotherapy**

Regimens of first-line chemotherapy	Overall	MTB or MK infection	
		(+)	(–)
	N = 30	N = 7	N = 23
Intensive regimens	26	6	20
FOLFOX	8	2	6
FOLFOX + Bmab	6	1	5
FOLFIRI	6	1	5
FOLFOX + Pmab/Cmab	2	2	0
FOLFIRI + Pmab/Cmab	2	0	2
FOLFIRI + Bmab	1	0	1
XELOX	1	0	1
Non-intensive regimens	4	1	3
UFT/LV	1	1	0
S-1	2	0	2
Capecitabine	1	0	1
Response rate (%)	40.0	28.6	43.5
CR	1	1	0
PR	11	1	10
SD	10	2	8
PD	7	3	4
NE	1	0	1

CR: complete response, PR: partial response, SD: stable disease, PD: progression disease, NE: not evaluable, RR: response rate.

FOLFOX: folinic acid, fluorouracil, oxaliplatin, Bmab: bevacizumab, FOLFIRI: folinic acid, fluorouracil, irinotecan, Pmab: panitumumab, Cmab: cetuximab, XELOX: capecitabine plus oxaliplatin, UFT/LV: uracil/tegafur/leucovorin.

### Second-line chemotherapy and others

Of 7 patients with MTB or MK infection, 4 received second-line chemotherapy after disease progression, whereas 2 with poor PS did not due to cancer death after first-line chemotherapy, and 1 with CR did not owing to the well-maintained CR. All 4 patients who received second-line chemotherapy achieved PR or SD, with 2 of them currently receiving fourth- and fifth-line chemotherapy, respectively.

Seventeen of 21 patients without MTB or MK infection received second-line chemotherapy, whereas 3 did not due to cancer death; 2 continued first-line chemotherapy with SD, and 1 was followed up only after discontinuation of first-line chemotherapy.

### Thoracic CT findings in m-CRC patients with MTB or MK infection

**Figure 1:**
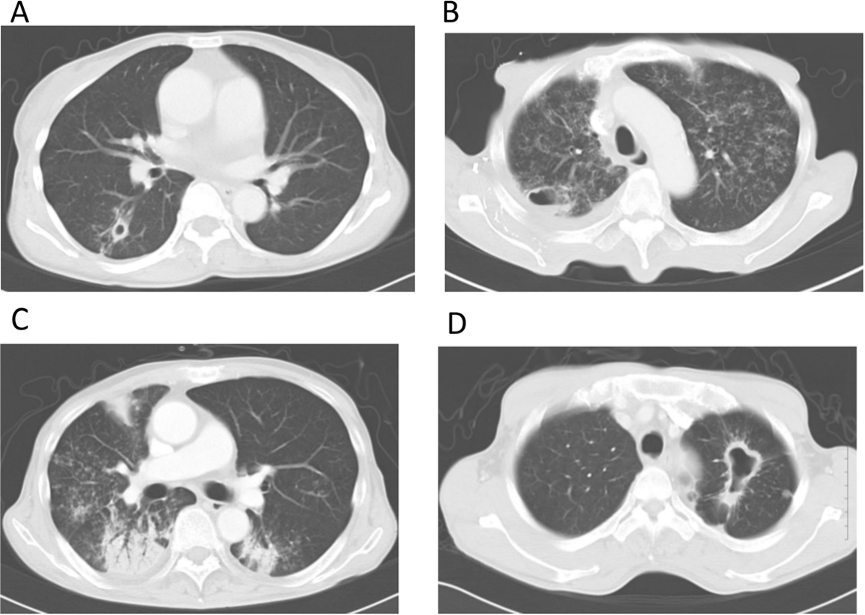
**Thoracic computed tomography findings in patients with MTB or MK infection. A:** Cavity formation with thick wall. **B:** Infiltration shadow in bilateral lungs and cavity formation in the right upper lobe. **C:** Infiltration shadow with patty follicular spot in the bilateral lungs. **D:** Cavity formation with thick wall in the left upper lobe.

Figure 1 shows thoracic CT findings in patients with MTB or MK infection. These CT images (A–D) presented a combination of cavity of the lung with thick or thin wall, infiltration shadows, and patty follicular spot.

### Diagnosis for MTB or MK infection in m-CRC patients

Table 3 shows the clinical features of 7 m-CRC patients with MTB or MK infection at the beginning of first-line chemotherapy. Of these, 2 were diagnosed by both sputum smear and culture for MTB, 2 by MTB-PCR and culture, 1 by MTB culture only, 1 by MK culture only, and 1 by CT imaging only.

**Table 3:** **Clinical outcomes of CRC patients with MTB or MK infection**

Patient	Diagnosis method for MTB or MK infection	Treatment of MTB or MK	Time from the beginning of MTB or MK therapy until the start of first-line chemotherapy (days)	MTB or MK treatment success
A	CT imaging	6HRE/6HR	16	Completion
B	Sputum TB-PCR (+), MTB culture (+)	2HREZ/7HR	104	Cure
C	Sputum smear (+), MTB culture (+)	24HE	408	Cure
D	Sputum smear (+), MK culture (+)	18HRE	56	Cure
E	Sputum smear (-), MTB culture (+)	9HRE	25	Cure
F	Sputum smear (-), Sputum TB-PCR (+), MTB culture (+)	6HRE/6HR	19	Cure
G	Sputum smear (+), MTB culture (+)	12RE	53	Cure
**Patient**	**First-line chemotherapy Regimen**	**Best response**	**Side effects (grade ≥ 3) due to cancer chemotherapy or MTB or MK treatment**	
A	UFT/LV	SD	-	
B	FOLFOX6 + Cmab	CR	-	
C	FOLFOX6	PD	Liver dysfunction due to MTB treatment	
D	FOLFOX6 + Pmab	PD	-	
E	FOLFOX6 + Bmab	SD	Hemoptysis due to Bmab	
F	FOLFOX6	PR	-	
G	FOLFIRI	PD	-	

MTB: Mycobacterium tuberculosis, MK: Mycobacterium kansasii, CT: computed tomography, TB-PCR: tuberculosis polymerase chain reaction.

H: isoniazid, R: rifampicin, E: ethambutol, Z: pyrazinamide.

nHREZ: n months of H, R, E and Z combination.

CR: complete response, PR: partial response, SD: stable disease, PD: progression disease.

FOLFOX: folinic acid, fluorouracil, oxaliplatin, Bmab: bevacizumab, FOLFIRI: folinic acid, fluorouracil, irinotecan, Pmab: panitumumab, Cmab: cetuximab, XELOX: capecitabine plus oxaliplatin, UFT/LV: uracil/tegafur/leucovorin.

### MTB or MK treatment success

Six of 7 patients with MTB or MK achieved a cure for their infection, whereas the other one completed the planned treatment with an improvement of chest CT imaging. None of these experienced MTB or MK infection relapses during or after cancer chemotherapy.

### Time to first-line chemotherapy from the beginning of MTB or MK therapy

The median time to first-line chemotherapy from the beginning of MTB or MK therapy was 53 days (range, 16 to 408 days). Patient C who waited 408 days received S-1 as adjuvant chemotherapy for gastric cancer for 6 months and concurrent MTB treatment during that time. After approximately 1 year, the patient was diagnosed with m-CRC and subsequently received CRC chemotherapy and additional MTB treatment for approximately 10 months.

### Side effects due to first-line chemotherapy or MTB treatment

Of 7 m-CRC patients with MTB or MK, patient C experienced grade-3 liver dysfunction due to rifampicin while receiving S-1 for post-operative adjuvant chemotherapy for gastric cancer. After the diagnosis of m-CRC, patient C received both cancer chemotherapy for CRC and a regimen containing HE and ofloxacin. Patient E who was treated with FOLFOX + Bmab as first-line chemotherapy experienced grade-3 hemoptysis after 2 cycles. After discontinuation of Bmab, the patient was treated safely with FOLFOX until PD.

### Overall survival and prognostic factors

**Figure 2:**
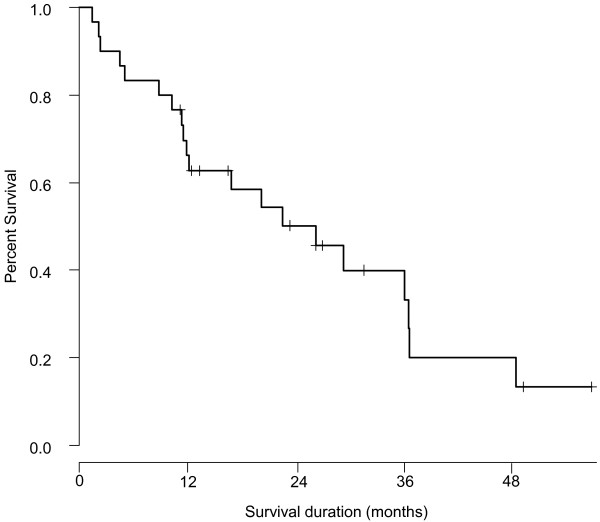
**Survival curve of 30 metastatic colorectal cancer patients.** The median survival time was 26.3 months (95% confidence interval: 11.2–48.6).

**Figure 3:**
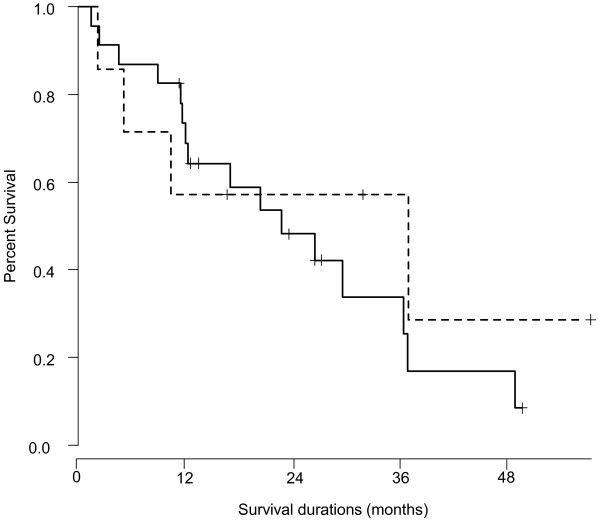
**Survival curve of colorectal cancer patients with MTB or MK and those without.** The median survival time of patients with MTB or MK infection and those without was 36.7 and 22.6 months, respectively. Dashed line indicates the survival curve of patients with MTB or MK, whereas solid line represents that of those without. No significant differences were observed between the two groups (P = 0.536).

As shown in Figure 2, the median survival time (MST) of all enrolled m-CRC patients was 26.3 months. Figure 3 shows the survival curves of patients with MTB or MK infection and those without, and the MST was 36.7 and 22.6 months, respectively. No significant survival differences were observed between both groups (P = 0.536). As shown in Table 4, PS and liver metastasis were identified as significant prognostic factors of survival by both univariate (P = 0.002 and 0.020, respectively) and multivariate (P = 0.004 and 0.030, respectively) analyses, whereas other factors were not significantly correlated with survival.

**Table 4:** **Univariate and multivariate analyses of overall survival (N = 30)**

Variable		N	MST	P value/HR (95% CI)	
			(Months)	Univariate	Multivariate
Sex					
	Male	20	29.4	0.458	
	Female	10	22.6	1.44 (0.54–3.81)	
Age					
	<69	14	29.4	0.569	
	≥69	16	22.6	1.30 (0.53–3.16)	
Performance status					
	0–1	19	36.1	0.002	0.004
	2–4	11	11.4	4.06 (1.58–10.43)	4.11 (1.57–10.76)
CEA (ng/ml)					
	<16.9	14	29.4	0.386	
	≥16.9	16	22.6	1.51 (0.59–0.82)	
K-ras					
	Wild-type	12	17.4	0.051	
	Mutated	14	36.7	0.357 (0.12–1.05)	
Stage					
	Relapse after surgery	15	36.7	0.453	
	IV	15	22.6	1.42 (0.56–3.59)	
Respiratory diseases					
	No	19	22.6	0.875	
	Yes	11	29.4	0.93 (0.37–2.34)	
MTB or MK infection					
	No	23	22.6	0.536	
	Yes	7	36.7	0.705 (0.23–2.15)	
Primary site					
	Rectum	16	29.4	0.322	
	Colon	14	22.6	0.615 (0.23–1.62)	
Number of metastatic organs					
	1	13	36.7	0.0893	
	≥2	17	16.9	2.2 (0.87–5.58)	
Lung metastasis					
	No	12	16.9	0.483	
	Yes	18	29.4	0.72 (0.29–1.79)	
Liver metastasis					
	No	17	36.1	0.020	0.030
	Yes	13	11.9	3.24 (1.15–9.11)	3.37 (1.13–10.07)

HR: hazard ratio, CI: confidence interval, MST: median survival time.

CEA: carcinoembryonic antigen, MTB: *Mycobacterium tuberculosis,* MK: *Mycobacterium kansasii*.

## Discussion

The present study indicate that patients with active MTB or MK infection could safely receive both cancer chemotherapy and effective MTB or MK treatment, and subsequently achieved a MST of 36.7 months, which was comparable to previously published results [23, 24]. Our findings therefore suggest that if effective MTB or MK treatment is provided, cancer chemotherapy could be administered in m-CRC patients with MTB or MK infection similarly to those without. However, although we achieved an overall RR of 40% for first-line chemotherapy, which was comparable to those previously reported 40–50% [23, 24], patients with MTB or MK infection had a lower RR of 28.9%. Possible explanations to such a result could be that 4 of 7 patients with MTB or MK infection received intensive regimens without molecular targeted agents or non-intensive regimen, and the other one who received FOLFOX + Bmab discontinued the treatment due to grade-3 hemoptysis after 2 cycles. Since patients with MTB or MK infection frequently have cavity lesions in the lung or expectorate hemosputum, they would have high risk for hemoptysis. Therefore, when grade-3 hemoptysis was observed, we decided not to provide Bmab-containing regimens to these patients to avoid hemoptysis death at first-line chemotherapy. Such a decision might lead to the low RR in patients with MTB or MK infection. However, despite a low RR to first-line chemotherapy, these patients achieved a MST of 36.7 months. Of 7 patients with MTB or MK infection, 4 received second-line or further chemotherapy to achieve SD or PR, whereas 1 maintained CR with first-line chemotherapy only. Except for the early deaths of 2 patients with poor PS, the remaining 5 experienced long-term survival.

As Falagas *et al.* [25] described in their review article, MTB and cancer are very common diseases but there has been little attention to the pathophysiological and practical implications of their co-existence. Most clinical studies selected in their review article [25] were case reports except for several case series and reviews, and all diagnoses were based on biopsies from the same site of infection and malignancy or from regional lymph nodes instead of acid fast bacterium culture. In this study, primary CRC lesions did not co-exist with MTB in the lungs, and each lesion was diagnosed separately. Furthermore, most cases examined in the review article [25] had received variable treatments for MTB prior to, concurrent with, or after cancer therapy. In our study, even if the patients expectorated sputum positive for MTB, they concurrently received cancer chemotherapy and MTB treatment after being diagnosed with both diseases. Since there is no study similar to ours, we confirmed in this study that m-CRC patients with MTB or MK should be able to safely and effectively receive cancer chemotherapy similar to those without.

Regarding MTB and cancer, previous studies reported that MTB was linked to the development of pyothorax associated lymphoma [26, 27] and lung cancer [28, 29]. However, there is no study available on the correlation between CRC development and MTB. Therefore, the co-existence of MTB and CRC in this study is thought to occur by chance.

Countries and areas such as Brazil, Eastern Asia, South-Eastern Asia, and India are those burdened with MTB infection [4]. In addition, a recent study [30] has shown that Brazil, China, the Republic of Korea and Japan have high incidences of CRC. The number of CRC patients with MTB may increase in these countries. However, no publication is available on the clinical courses and cancer chemotherapy outcomes of CRC patients with MTB infection.

To the best of our knowledge, this study was the first to demonstrate that m-CRC patients with active MTB or MK infection were able to safely and effectively continue both cancer chemotherapy and MTB or MK treatment concurrently, and did not experience relapse of MTB or MK infection during their total clinical courses after successful MTB or MK treatment.

The limitations of this study included its retrospective design and small cohort of patients. Thus, it remains unclear whether its results would be generalized for other solid tumors or at other institutions.

## Conclusions

Our results indicate that m-CRC patients with MTB or MK should be able to safely and effectively continue cancer chemotherapy to subsequently achieve comparable survival duration to those without the infection if they receive proper MTB or MK treatment. As a result, we plan to prospectively examine the safety and efficacy of cancer chemotherapy and concurrent MTB treatment in patients with CRC or other solid tumors having active MTB infection.
